# Seed Germination Ecology of the Medicinal Plant *Peganum harmala* (*Zygophyllaceae*)

**DOI:** 10.3390/plants12142660

**Published:** 2023-07-16

**Authors:** Shifeng Li, Ning Yan, Mohsin Tanveer, Zhenyong Zhao, Li Jiang, Hongling Wang

**Affiliations:** 1 College of Water Resources and Civil Engineering, China Agricultural University, Beijing 100083, China; lsf96504@cau.edu.cn; 2 Planning and Design Institute, China Agricultural University, Beijing 100083, China; yanning98@126.com; 3 State Key Laboratory of Desert and Oasis Ecology, Xinjiang Institute of Ecology and Geography, Chinese Academy of Sciences, Urumqi 830011, China; mohsin.tanveer@utas.edu.au (M.T.); zhaozhy@ms.xjb.ac.cn (Z.Z.); 4 Tasmanian Institute of Agriculture, University of Tasmania, Hobart, TAS 7001, Australia; 5 CAS Research Center for Ecology and Environment of Central Asia, Xinjiang Institute of Ecology and Geography, Chinese Academy of Sciences, Urumqi 830011, China; 6 Bayinbuluk Grassland Ecosystem Research Station, Xinjiang Institute of Ecology and Geography, Chinese Academy of Sciences, Bayinbuluk 841314, China

**Keywords:** drought, salinity, seed germination, *Peganum harmala*, photoperiods, temperature

## Abstract

Seed germination is a crucial stage in the life cycle of annuals in arid, saline regions and is particularly vulnerable to abiotic stresses. *Peganum harmala*, a valuable medicinal plant, has limited research on its seed germination response to different environmental stresses in the arid, saline regions of Central Asia. To investigate this, we studied the effects of various temperature regimes (ranging from 20/5 to 35/20 °C), light exposure (12 hours light/12 hours dark and continuous dark), seven levels of polyethylene glycol (PEG-6000) concentration (ranging from 0–30%), and four types of salinity (ranging from 0–600 mmol L^−1^). Our findings show that photoperiod and temperature significantly influence germination. Optimal temperature range for seed germination was observed at 30/15 °C, with simulated critical and limit values of drought tolerance being highest (17.30% and 24.98%). However, higher temperatures (35/20 °C) and lower temperatures (20/5 °C) reduced the critical and limit values of drought tolerance. Additionally, the type and concentration of salinity had a significant effect on the seed germination, shoot, and root lengths of *P. harmala*. Regression analysis indicated that the critical values of NaCl, Na_2_SO_4_, NaHCO_3_, and Na_2_CO_3_ tolerance during germination were 178 mmol L^−1^, 101 mmol L^−1^, 106 mmol L^−1^, and 54 mmol L^−1^, respectively. Salinity inhibition on seed germination followed the order: NaCl < NaHCO_3_ < Na_2_SO_4_ < Na_2_CO_3_. Moreover, NaCl, Na_2_SO_4_, NaHCO_3_, and Na_2_CO_3_ significantly inhibited the growth of *P. harmala* seedlings in both shoots and roots. Our study demonstrates the sensitivity of *P. harmala* to environmental factors such as light, temperature, drought, and salinity. The study provides valuable information on the germination ecology of *P. harmala* under diverse ecological scenarios, which can be useful in developing efficient propagation and utilization of this medicinal plant.

## 1. Introduction

Medicinal plants have played a crucial role in human civilization since ancient times and continue to play a crucial role today [1]. These plant species possess powerful medicinal properties that can treat a wide range of ailments and provide valuable nutritional benefits. Additionally, they make significant contributions to human well-being and economic development [2,3]. One such plant is *Peganum harmala*, commonly known as Syrian Rue. This herbaceous perennial plant belongs to the family *Zygophyllaceae* and is native to Central Asia and the Mediterranean region [4]. It is often grown in arid and semi-arid lands, and its seeds are widely used in traditional medicine to treat various ailments such as gastrointestinal, respiratory, and nervous system disorders [5].

Seed germination is an essential stage in the life cycle of plants. During the process of germination, stored nutrients like lipids, carbohydrates, and proteins are used up. At the same time, the cells grow larger by stretching, resulting in an increase in their size [6,7]. It is greatly influenced by various ecological factors such as temperature, light, water availability, and soil salinity [8,9]. These factors significantly influence the quality and quantity of seeds produced and their subsequent germination. Furthermore, they can affect the growth and development of young seedlings, which ultimately contributes to the successful establishment of the plant in its native or introduced range [10,11]. Thus, it is imperative to understand and analyze the effects of these ecological factors on the seed germination and seedling growth of *P. harmala* to ensure successful propagation and utilization in medicinal practices.

Light is a critical factor that affects seed germination process. Light can affect germination through photoreceptor-mediated responses. There is significant variability in the light requirement for seed germination [12,13]. For instance, while the germination of eight out of 16 subtropical halophytes was stimulated in the presence of light, seeds of two species germinated optimally in darkness, and six species were not affected by light [14]. The germination of small seeds is frequently dependent on light [15]. *P. harmala* seeds have been reported to have a light-dependent germination ability [16], which means that they require light to start the germination process. However, the effect of light on the germination of *P. harmala* seeds at different temperatures is unclear.

Temperature is another important factor that affects plant growth, development, and seed germination [17]. Seed germination is generally faster at warmer temperatures, and the optimal temperature range for germination varies among species and populations [18,19]. In arid and semi-arid regions, the temperature fluctuates greatly between day and night. High temperatures during the day and low temperatures at night create a thermal fluctuation that can affect the germination of seeds. Studies have shown that *P. harmala* seeds have higher germination percentages when exposed to high temperatures [16]. However, the specific temperature range that is optimal for *P. harmala* seed germination is still unclear.

Drought is a significant abiotic factor that affects plant germination and seedling development in arid and semi-arid regions [20,21]. Polyethylene glycol (PEG) is a synthetic compound that is not present in nature; however, it has been extensively used in scientific research to simulate drought conditions. The effects of PEG on seeds have been extensively studied to understand the physiological responses and adaptive mechanisms of plants during water scarcity. PEG treatment is considered an effective method for inducing water stress in seeds, mimicking the drought conditions experienced in nature. By exposing seeds to PEG, researchers can examine the effects of a water deficit on germination, seedling growth, and various other biochemical and molecular processes [22,23]. One of the main ways in which drought impacts seed germination is by reducing the availability of water needed for the process [24]. In such environments, water availability is often limited to short periods, making it crucial for vegetation restoration efforts to focus not only on achieving rapid and uniform seed germination but also on ensuring that seeds can germinate successfully under low water availability conditions [25,26]. For instance, *P. harmala* seeds have been found to be able to germinate within a water potential range of 5% to 20% PEG, indicating their high tolerance for drought stress [27]. However, research is lacking on the germination performance of *P. harmala* seeds under different temperature regimes and different degrees of drought stress. Therefore, it is essential to further investigate the effects of these factors on seed germination to better inform successful vegetation restoration efforts.

As the germination process takes place, soil salinity can disrupt the delicate balance of nutrients and hormones (such as gibberellin and abscisic acid) necessary for growth. High salinity levels can lead to delayed or inhibited germination, depending on the plant’s tolerance to salt stress. Salt stress can affect all stages of plant growth and development, leading to nutrient imbalances as plants experience both osmotic and nutrient stress in saline soil environments. The impact of this salinity-induced stress on seed germination and plant growth can vary greatly depending on factors such as the type and amount of salt present and the prevailing climatic conditions, particularly in arid regions with low precipitation, and high evapotranspiration rates. Over 6% of the world’s total land area, totaling more than 800 million hectares, is affected by salt-affected soil. Such soil is characterized by high concentrations of soluble minerals such as sodium as well as anions like sulfate, chloride, carbonate, and bicarbonate (Cl^−^, SO_4_^2−^, CO_3_^2−^, and HCO_3_^−^). The availability of these salts varies across different arid and semi-arid soils around the world. The ability of plant species to survive and reach maturity in salt-affected soil depends on their tolerance to salt stress, which can differ based on the species and growing conditions. The development and screening of salt-tolerant seed varieties is therefore crucial for utilizing saline soil effectively. Existing research mainly focuses on evaluating seed germination with sodium chloride (NaCl), and limited data is available on how different soluble salts affect the germination of medicinal and aromatic plant species. Further research is necessary to understand the germination performance of *P. harmala* seeds under different temperatures and levels of drought stress. While previous research on plant salinity stress primarily uses NaCl as a simulation [28], only a few studies explore other forms of alkaline salinity stress, such as NaHCO_3_ and Na_2_CO_3_. These forms of stress pose the dual challenges of salinity ion saturation and high soil pH levels. *P. harmala* seeds have been found to exhibit high salt tolerance, germinating under salinity concentrations ranging from 0–200 mmol L^−1^. Nonetheless, it is essential to investigate the germination performance of *P. harmala* seeds under other forms of salinity stress to better understand their salt tolerance.

While the chemical composition of *P. harmala* has been extensively studied in the past [29,30,31], little attention has been paid to its seed germination and seedling growth. Therefore, this research aims to evaluate the impact of various environmental factors, such as light, temperature, drought, and salinity, on seed germination. Specifically, we aim to (i) determine the effects of a wide range of temperature and light conditions on germination, including their interaction; (ii) assess the effects of temperature and drought on germination and early seedling development; and (iii) evaluate the impact of different types and concentrations of salinity on seed germination and seedling growth. The findings of this study could assist in improving the efficacy of seed germination and contribute to the broader field of plant ecology. Additionally, the results could help medicinal plant cultivators develop sustainable practices that enhance conservation and ecological diversity.

## 2. Results

### 2.1. Seed Characteristics

**Figure 1:**
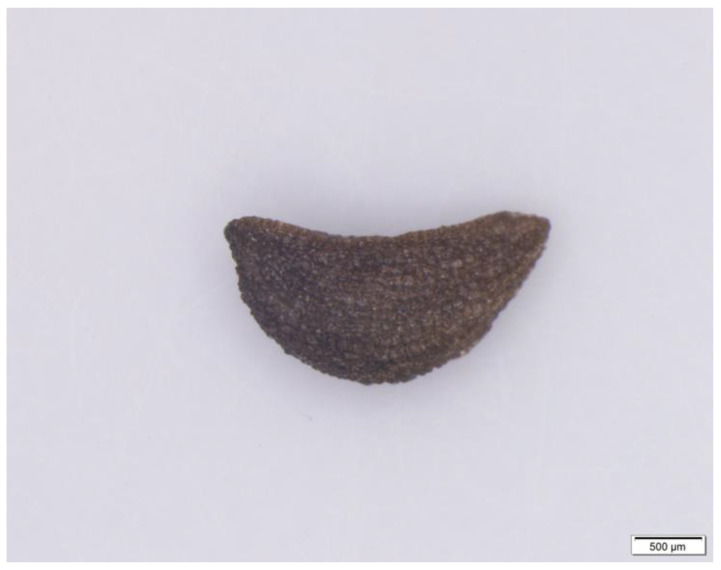
Morphology of *Peganum harmala* seed under a stereomicroscope.

The seeds of *P. harmala* are typically dark brown, slightly curved in shape, and 3-angulate. The surface of seeds is muriculate (Figure 1). The length of the seeds is 1.78 ± 0.17 mm. The mass of seeds is 0.2007 ± 0.0016 g per 100 seeds.

### 2.2. Imbibition Test

**Figure 2:**
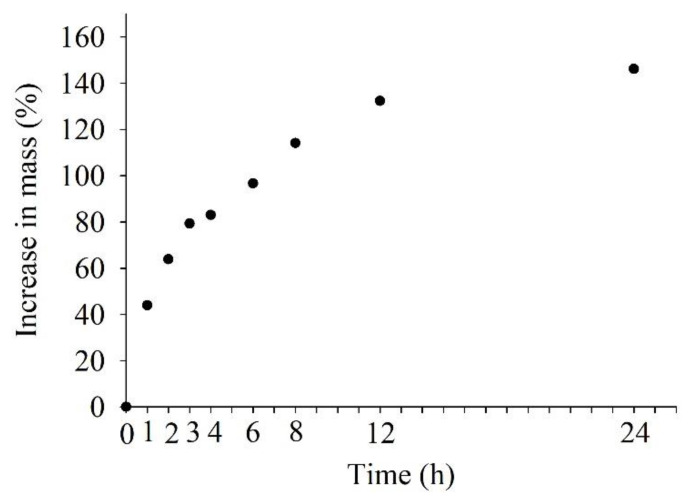
Imbibition curves for *Peganum harmala* seed in distilled water.

*Peganum harmala* seeds imbibed water readily and followed a typical pattern of rapid initial water uptake, with seed mass increasing by 43.9 + 4.3% after 1 h, 83.0 + 3.4% after 4 h, and 114.1 + 3.2% after 8 h (Figure 2).

### 2.3. Effects of Light and Temperature on Germination

**Figure 3:**
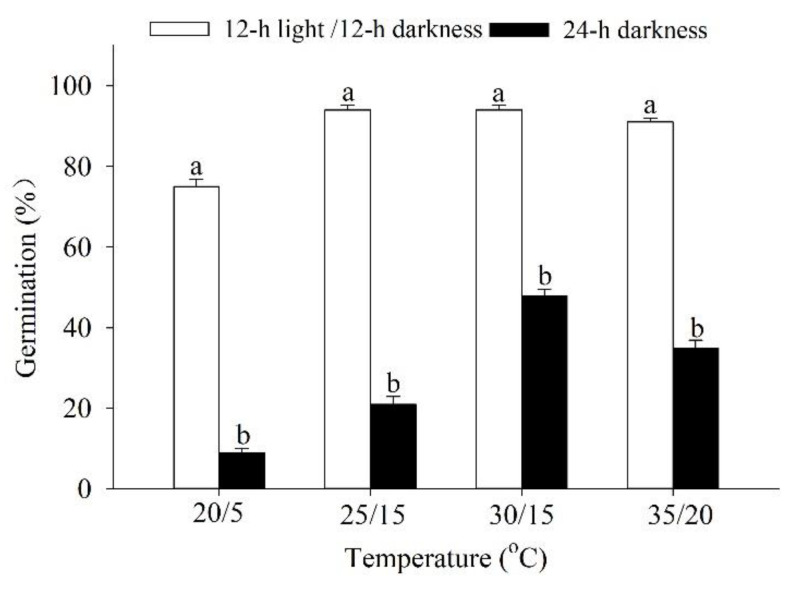
Effects of light and temperature on germination of *Peganum harmala.* Values with different lowercase letters on the bars indicate significant differences (*p* < 0.05) between the same temperature treatments.

The germination of *P. harmala* seeds was significantly influenced by temperature (*p* < 0.01), light (*p* < 0.01), and their interaction (*p* < 0.01). Optimal germination performance was observed at 25/10 °C (94%) and 30/15 °C (94%) for two light regimes (light/dark-12 h/12 h, dark-24 h), while the worst performance was observed at 20/5 °C (9%). There was a significant effect of light on seed germination of *P. harmala* across all test temperatures (Figure 3).

### 2.4. Effects of Drought and Temperature on Germination and Seedling Growth

**Figure 4:**
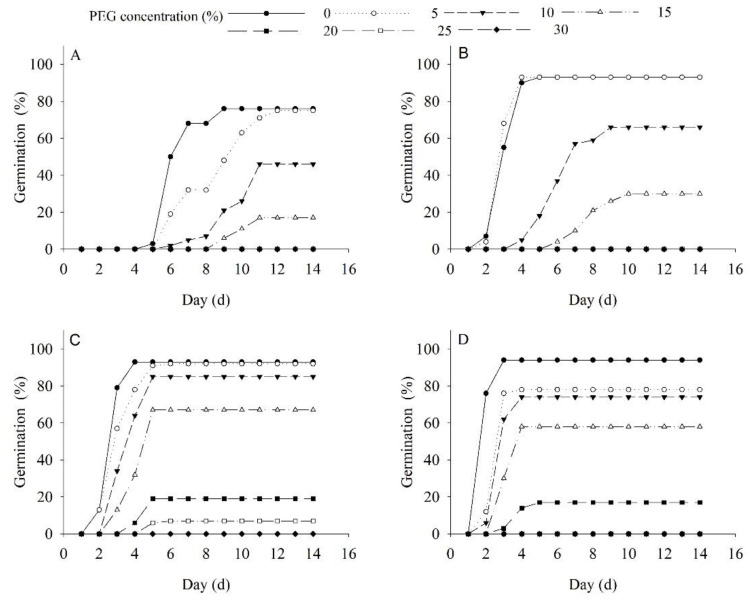
Effects of drought on germination of *Peganum harmala* under (**A**) 20/5 °C, (**B**) 25/10 °C, (**C**) 30/15 °C, and (**D**) 35/20 °C treatments.

The germination of *P. harmala* seeds was significantly influenced by PEG concentration (*p* < 0.01), temperature (*p* < 0.01), and their interaction (*p* < 0.01). Only the germination at 35/20 °C significantly decreased (by 17.02%) under 5% PEG treatment, whereas there was no significant decrease under 20/5 °C, 25/10 °C, and 30/15 °C treatments. Higher temperatures were found to reduce the drought tolerance of *P. harmala* seeds. Under 10% PEG treatment, the germination only decreased significantly at 30/15 °C (Figure 4).

Regression analysis of germination showed a significant negative correlation with PEG concentration for all four temperature treatments (20/5 °C, 25/10 °C, 30/15 °C, and 35/20 °C). The drought tolerance critical values and limit values were calculated for each temperature treatment (Table 1). For example, the critical values and limit values for 30/15 °C and 35/20 °C were calculated as 14.48% and 17.30%, 24.98%, and 24.48%, respectively (Table 1).

**Figure 5:**
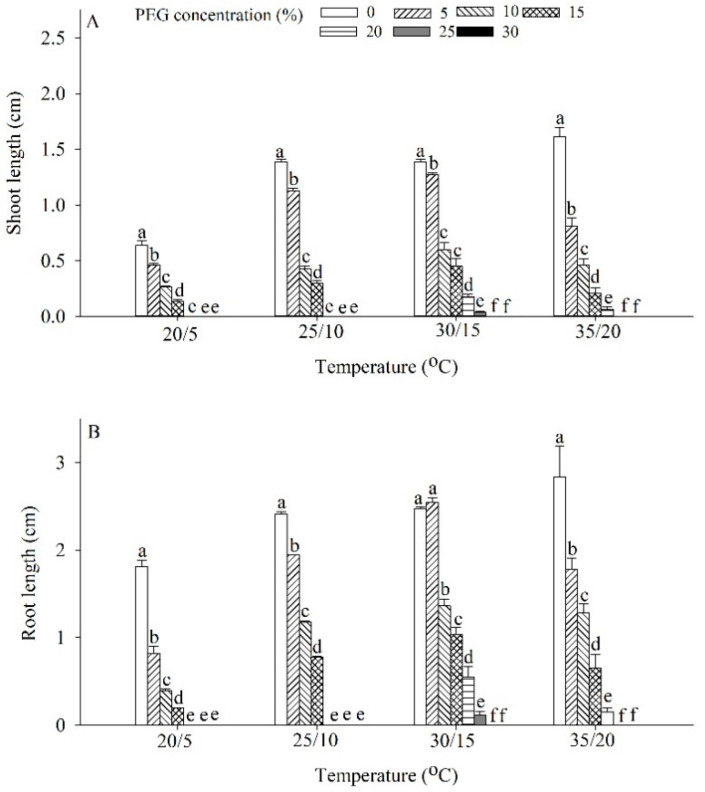
Effects of drought on (**A**) shoot and (**B**) root lengths of *Peganum harmala* under 20/5 °C, 25/10 °C, 30/15 °C, and 35/20 °C treatments. Values with different lowercase letters on the bars represent drought treatments under the same temperature that are significantly different at *p* < 0.05.

Both shoot and root lengths of *P. harmala* were significantly influenced by PEG concentration (*p* < 0.01), temperature (*p* < 0.01), and their interaction (*p* < 0.01). The shoot length under the 5% PEG treatment significantly decreased under all four temperature treatments. The root length significantly decreased under all four temperature treatments under 5% PEG treatment, except for 25/10 °C. Compared with the control, under 20/5 °C, 25/10 °C, 30/15 °C, and 35/20 °C, the root length of *P. harmala* seedlings treated with 10% PEG decreased significantly by 58.82%, 69.37%, 56.76%, and 71.32%, respectively; under 20/5 °C, 25/10 °C, 30/15°C, and 35/20 °C, the root length of *P. harmala* seedlings treated with 10% PEG decreased significantly by 78.62%, 51.30%, 49.94%, and 54.93%, respectively (Figure 5).

### 2.5. Effects of Salinity on Germination and Seedling Growth

**Figure 6:**
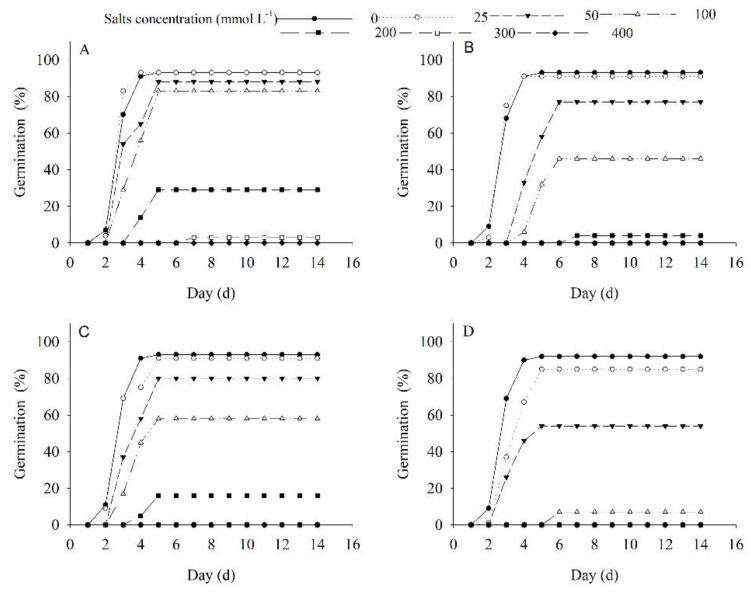
Effects of (**A**) NaCl, (**B**) Na_2_SO_4_, (**C**) NaHCO_3_, and (**D**) Na_2_CO_3_ on germination of *Peganum harmala*.

The germination of *P. harmala* is significantly affected by salinity concentration (*p* < 0.01), type (*p* < 0.01), and their interaction (*p* < 0.01). As the concentrations of NaCl, Na_2_SO_4_, NaHCO_3_, and Na_2_CO_3_ increase, seed germination is significantly reduced. At a lower concentration (100 mmol L^−1^ Na_2_CO_3_), the germination of *P. harmala* decreased significantly by 7.60%, while the same concentration of NaHCO_3_, NaCl, and Na_2_SO_4_ did not reduce germination significantly. At 200 mmol L^−1^ NaCl, Na_2_SO_4_, NaHCO_3_, and Na_2_CO_3_, the germination of *P. harmala* is 10.75%, 50.54%, 37.63%, and 92.39%, respectively (Figure 6).

A significant negative correlation with the concentrations of NaCl, Na_2_SO_4_, NaHCO_3_, and Na_2_CO_3_. The salinity tolerance critical values and limiting values of *P. harmala* seeds for NaCl, Na_2_SO_4_, NaHCO_3_, and Na_2_CO_3_ at the germination stage were calculated. The results indicated that the critical values are 177.97 mmol L^−1^, 100.85 mmol L^−1^, 106.27 mmol L^−1^, and 54.38 mmol L^−1^, respectively; The limit values are 299.21 mmol L^−1^, 206.78 mmol L^−1^, 230.34 mmol L^−1^, and 110.34 mmol L^−1^, respectively (Table 2). The order of salinity tolerance of *P. harmala* was Na_2_CO_3_ < Na_2_SO_4_ < NaHCO_3_ < NaCl.

**Figure 7:**
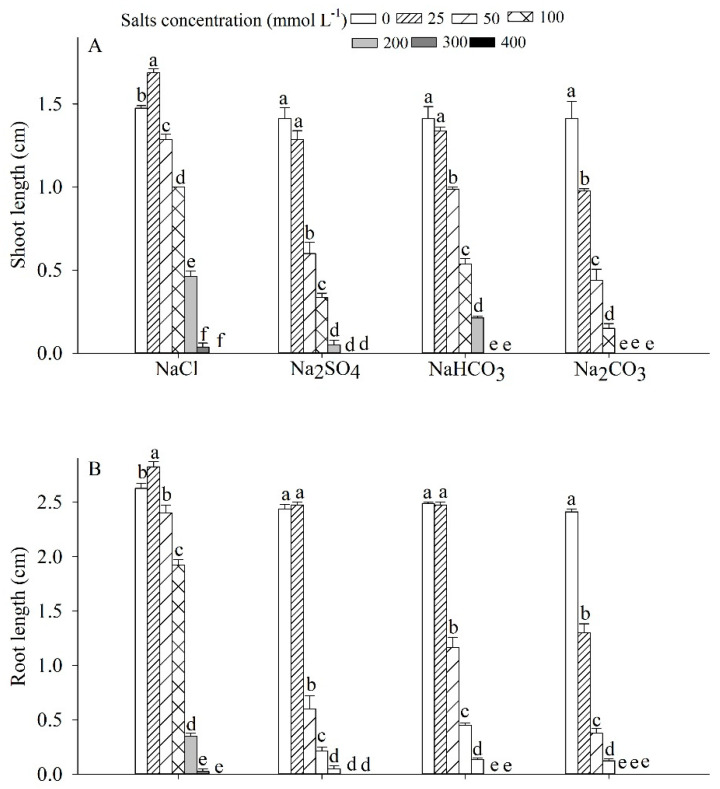
Effects of NaCl, Na_2_SO_4_, NaHCO_3_ and Na_2_CO_3_ on (**A**) shoot and (**B**) root lengths of *Peganum harmala*. Values with different lowercase letters on the bars represent salinity concentration treatments that are significantly different at *p* < 0.05.

Salinity concentration and type significantly affected the shoot and root growth of *P. harmala*. 25 mmol L^−1^ NaCl significantly promoted seedling shoot and root growth, while 25 mmol L^−1^ Na_2_CO_3_ significantly inhibited their growth. Under the treatment of higher concentrations of NaCl, Na_2_SO_4_, NaHCO_3_, and Na_2_CO_3_ (200 mmol L^−1^), the shoot and root of *P. harmala* significantly decreased; the seedling shoot length decreased by 32.20%, 76.11%, 69.95%, and 89.38%, respectively, and the root length decreased by 26.67%, 91.28%, 81.91%, and 94.82%, respectively. The inhibition degree of different types of salinity on seedling growth could be ranked in order: NaCl < NaHCO_3_ < Na_2_SO_4_ < Na_2_CO_3_ (Figure 7).

## 3. Discussion

Based on the high salt tolerance, reclamation potential, and medicinal value of *P. harmala* [28,29,30,31,32,33,34,35], this study investigated the germination responses of its seeds to various environmental factors, including light, temperature, drought, and salinity. Our findings suggest that *P. harmala* is sensitive to temperature, light, drought, and salinity. However, temperature significantly affects the drought tolerance of *P. harmala* during germination. The varying levels of salinity have different effects on inhibition on germination and seedling growth. Specifically, NaCl has the least inhibition, followed by NaHCO_3_, Na_2_SO_4_, and Na_2_CO_3_, in descending order of decreasing inhibitory effects.

Although light can be an important factor that influences the seed germination process, it is not a necessary prerequisite for regulating seed germination [12]. Previous studies have shown that the germination process of halophyte seeds can be sensitive to light [14]. In the present study, we investigated the role of light on the germination of *P. harmala* seeds. Our results demonstrate that *P. harmala* seeds are influenced by light for their germination requirements. Germination was significantly different between seeds exposed to light and those kept in darkness, possibly due to their thicker seed coat, indicating that light can be an important ecological regulation factor for the germination of *P. harmala* seeds, which may be related to the thin seed coat of *P. harmala*, that easily absorbs light signals, making some unique photosensitive enzymes quickly activated during the seed germination process. Additionally, when studying the effect of light exposure on *P. harmala* seeds, it was found that germination decreased significantly when the seeds were kept in complete darkness. However, when exposed to a light/dark cycle of 12 h each, germination percentages exceeded 90%. Previous research has also documented the specific light conditions required for *P. harmala* germination [27]. These findings indicate that in order to effectively manage weed infestations caused by *P. harmala*, it is necessary to either bury the seeds deeply through tillage or transition to conservation agricultural systems. By implementing conservation agricultural systems, a thick layer of crop residue cover can be established, which would provide shade and limit the amount of light reaching the seeds, ultimately reducing weed infestation in modern farming practices. Therefore, light is an ecological factor that affects the germination process of *P. harmala* seeds. This study explored the temperature adaptation range of *P. harmala* seeds during the stages of germination and seedling growth. Our investigations indicated that the optimal temperature conditions for seed germination of *P. harmala* were found to be 30/15 °C. *P. harmala* demonstrated a narrow temperature adaptation range, with low temperatures (20/5 °C, 25/10 °C) inhibiting germination and high temperatures (35/20 °C) negatively impacting seedling root growth. Moreover, *P. harmala* was found to be particularly vulnerable to low-temperature stress. The findings of this study shed light on the critical role that temperature sensitivity plays in the germination and population renewal of *P. harmala*, which could be useful for conservation efforts for this important species.

To evaluate the drought resistance of plant species, it is necessary to examine key parameters [36,37]. This study employed PEG to evaluate the drought resistance of *P. harmala* seeds during germination and the seedling stages. The concentration of PEG required to simulate a specific level of drought can vary depending on the specific experimental setup and the desired intensity of water scarcity. Generally, higher concentrations of PEG in the range of 15–30% are commonly used to induce severe drought conditions, while lower concentrations in the range of 5–15% represent moderate drought stress. Concentrations below 5% may be used to mimic mild water deficit conditions. PEG induces a water deficit by decreasing the water potential of the growth medium, which restricts water uptake by seeds or plants. This osmotic stress triggers a range of physiological and biochemical changes [22,23]. The research findings revealed that increasing PEG concentration reduced the germination rate, root length, and shoot length of *P. harmala*. However, mild drought stress during the 5% PEG treatment led to an increase in root biomass, suggesting an adaptive strategy for survival under limited nutrient resources. In addition to water availability, temperatures also influence the drought resistance of seeds and seedlings. The optimal conditions for *P. harmala* seed germination and seedling growth were found to be a temperature range of 30/15 °C and a 5% PEG treatment. These findings highlight the adaptability of *P. harmala* seeds to changes in desert environments and their ability to take advantage of short-term rainfall or floods for quick germination. The complexity of seed germination ecology necessitates considering multiple ecological factors when assessing plant drought resistance.

This study explored the impact of various types of salinity stress on the germination and seedling growth of *P. harmala*. Salinity and alkaline stress were both found to significantly inhibit seed germination, with the degree of inhibition dependent on the salinity concentration and type of salinity. The inhibitory effects of salinity stress on seed germination were primarily attributed to osmosis and ion toxicity, while alkali stress inhibited germination through higher pH values leading to ion imbalance in seeds [38,39]. Salt induces oxidative and osmotic stress, causing disruption of cell elongation and division [40,41]. Our findings indicate that the impact of neutral and low-concentration alkaline salinity on the growth of *P. harmala* seedlings was primarily observed in the shoot length, while salinity induced by a high concentration of NaHCO_3_ and the mixed salinity treatment showed a greater effect on root length. Interestingly, low-concentration salinity stress was found to promote seed germination due to slight osmotic stress. As the salinity solution concentration increased, the inhibitory effects became more severe. The study found that neutral salinity is less toxic to plant seeds than alkaline salinity, and root length was deemed a more appropriate metric for evaluating *P. harmala*’s salinity tolerance. These findings provide valuable insights into the mechanisms underlying the effects of salinity stress on seed germination and seedling growth. Additionally, they can be used to inform strategies for improving plant growth in saline-alkali environments.

## 4. Conclusions

In conclusion, our study provides valuable insights into the germination response of *P. harmala* in the arid, saline regions of Central Asia. Temperature was found to be crucial for seed germination, with an optimal range of 30/15 °C. We also observed the significant effect of photoperiod on germination. Furthermore, *P. harmala* demonstrated sensitivity to drought and salinity stress, with varying effects based on salt type and concentration. NaCl, Na_2_SO_4_, NaHCO_3_, and Na_2_CO_3_ concentrations significantly affected germination and seedling growth. Our findings highlight the importance of considering these stress factors for effective management practices in reconstructing arid and saline regions. Successful cultivation and management of *P. harmala* require understanding temperature, photoperiod, drought, and salinity. These findings can guide the development of strategies for the resilient cultivation and utilization of *P. harmala* in the medicinal plant industry. Future research can explore the ecological and economic significance of *P. harmala*’s response to environmental stresses, further enhancing its sustainable utilization.

## 5. Materials and Methods

### 5.1. Study Species

*Peganum harmala*, commonly known as Syrian Rue, is a perennial herbaceous plant that grows 30–70 cm tall and is much branched. Its roots can reach up to 2 cm in diameter. The leaves are alternate and ovate, divided into 3–5 linear to lanceolate-linear lobes. The flowers appear opposite the leaves on the upper parts of the branches. The sepals are divided into linear lobes, while the petals are yellowish-white and obovate-oblong. The plant, as a halophyte, prefers slightly saline sands near oases and dry grasslands in desert areas, thriving at altitudes between 400 and 3600 m. This plant species is distributed across Central Asia, as well as in some areas of North Africa, Western Asia, and Southern Europe. *P. harmala* is also considered a noxious weed that frequently escapes cultivation in desert areas worldwide.

### 5.2. Seed Collection

In October 2021, freshly matured fruits of *P. harmala* were collected from a natural population (45°26.2577′ N, 85°00.3979′ E) in a saline desert (pH 8.7, soil salinity content 1.6%) in northern Xinjiang, China. In the laboratory, we manually removed the seeds from the dry fruit by rubbing. Subsequently, the seeds were stored at room temperature until their utilization in the experiment pertaining to seed germination ecology.

### 5.3. Seed Characteristics

The size, shape, and color of *P. harmala* seeds were recorded using a stereomicroscope (Olympus SZX10, Tokyo, Japan). Four groups of 100 seeds were weighed using an analytical balance (precision 0.0001 g).

### 5.4. Imbibition Test

An imbibition test was conducted at room temperature (23 ± 2 °C) using four replicates of 25 dry seeds. Seeds were placed in 50-mm-diameter Petri dishes with distilled water-moistened filter paper. Furthermore, seed mass was measured at time 0 and after 1, 2, 3, 4, 6, 8, 12, and 24 h. The relative increase in fresh weight (Wr) of seeds was calculated as Wr = [(Wf – Wi)/ Wi] × 100, where Wi is the initial seed weight and Wf is the weight after a certain time.

### 5.5. Effects of Light and Temperature on Germination

We used an alternating 12-h light (3000 Lx) and 12-h darkness cycle of 20/5, 25/10, 30/15, and 35/20 °C. We set 25 seeds in each Petri dish (5 cm in diameter), placed them on a double layer of filter paper, and moistened them with 2.5 mL of distilled water. We subjected the seeds to the different temperature regimes in light and darkness (with the Petri dishes wrapped in two layers of aluminum foil), and there were four biological replicates for each treatment. A seed was considered to have germinated when the emerging radicle measured at least 1 mm. We observed the germination once a day for 14 days and checked the seeds incubated in darkness at the end of the experiment.

### 5.6. Effects of Drought and Temperature on Germination and Seedling Growth

To investigate the effect of different polyethylene glycol (PEG-6000) concentrations, seven concentrations (0%, 5%, 10%, 15%, 20%, 25%, and 30%) were utilized. The samples were incubated at various temperatures ranging from 20/5 to 35/20 °C while maintaining a 12-h temperature cycle for 14 days. Daily observations were recorded of the germination process. After the experiment, shoot and root lengths were measured using a rectilinear scale. Shoot and root lengths of *P. harmala* seedlings were measured using a rectilinear scale (mm).

### 5.7. Effects of Salinity on Germination and Seedling Growth

To study the effects of salt concentrations commonly found in local salt soil on seed germination, we prepared saline solutions using NaCl, Na_2_SO_4_, NaHCO_3_, and Na_2_CO_3_ at equimolar concentrations ranging from 0 to 400 mmol L^−1^. Petri dishes containing seeds were maintained at a variable temperature of 25/10 °C under a 12-h light/12-h dark cycle. Daily observations were recorded for the number of germinated seeds, and the shoot and root lengths of the germinated seeds were measured at the end of the experiment. Shoot and root lengths of *P. harmala* seedlings were measured using a rectilinear scale (mm).

### 5.8. Data Analysis

We conducted various analyses using SPSS Version 21.0 (SPSS Inc., Chicago, IL, USA). To investigate the effects of light and temperature on the germination of *P. harmala* seeds, we used a two-way ANOVA. Similarly, we used a two-way ANOVA to examine the effects of drought and temperature on the germination, shoot, and root lengths of P. harmala seeds. Furthermore, we analyzed the effects of different salinity types and concentrations on the germination, shoot, and root lengths of *P. harmala* seeds using a two-way ANOVA. For each treatment, we used one-way ANOVA and Tukey’s test to detect significant differences. Finally, we used linear regressions to establish the relationships between germination and different concentrations of PEG or salinity.

## Figures and Tables

**Table 1:** The regression analysis of PEG concentrations with germination at different temperatures.

Temperature	Regression Equation	Correlation Coefficient	Critical Value PEG (%)	Limit Value PEG (%)
20/5 °C	y = −914.2857x^2^ − 237.1429x + 80.2288	0.9592	9.37%	19.37%
25/10 °C	y =−1971.4286x^2^ − 103.7143x + 96.3429	0.9745	14.48%	19.63%
30/15 °C	y = −1564.2857x^2^ + 9.9286x + 95.1071	0.9377	17.30%	24.98%
35/20 °C	y =−1092.8571x^2^ − 109.0714x + 92.1786	0.9578	15.28%	24.48%

**Table 2:** The regression analysis of salinity concentrations for four types of salinity and germination.

Salinity Type	Regression Equation	Correlation Coefficient	Critical Value (mmol L−^1^)	Limit Value (mmol L^−1^)
NaCl	y = −0.0004x^2^ − 0.2022x + 97.8060	0.9847	177.97	299.21
Na_2_SO_4_	y = −4720x + 97.6000	0.9673	100.85	206.78
NaHCO_3_	y = −0.4030x + 97.8250	0.9646	106.27	230.34
Na_2_CO_3_	y = −0.8937x + 98.6000	0.9649	54.38	110.32

## Data Availability

The data presented in this study are available on request from the corresponding author.
